# Structural and thermodynamic basis of proline-induced transmembrane complex stabilization

**DOI:** 10.1038/srep29809

**Published:** 2016-07-20

**Authors:** Thomas Schmidt, Alan J. Situ, Tobias S. Ulmer

**Affiliations:** 1 Department of Biochemistry & Molecular Biology and Zilkha Neurogenetic Institute, Keck School of Medicine, University of Southern California, 1501 San Pablo Street, Los Angeles, CA 90033, USA.

## Abstract

In membrane proteins, proline-mediated helix kinks are indispensable for the tight packing of transmembrane (TM) helices. However, kinks invariably affect numerous interhelical interactions, questioning the acceptance of proline substitutions and evolutionary origin of kinks. Here, we present the structural and thermodynamic basis of proline-induced integrin αIIbβ3 TM complex stabilization to understand the introduction of proline kinks in membrane proteins. In phospholipid bicelles, the A711P substitution in the center of the β3 TM helix changes the direction of adjacent helix segments to form a 35 ± 2° angle and predominantly repacks the segment in the inner membrane leaflet due to a swivel movement. This swivel repacks hydrophobic and electrostatic interhelical contacts within intracellular lipids, resulting in an overall TM complex stabilization of −0.82 ± 0.01 kcal/mol. Thus, proline substitutions can directly stabilize membrane proteins and such substitutions are proposed to follow the structural template of integrin αIIbβ3(A711P).

In the evolution of globular proteins, structural complexity and functionality can be increased by combining independently folding protein domains^1^,^2^. In contrast, in membrane proteins, individual intramembraneous domains are not apparent beyond transmembrane (TM) helices and an increase in complexity necessitates an increase in the overall number of TM helices. In the human genome, multi-pass (polytopic) membrane proteins are predicted to exhibit an average number of 6.6 TM helices and to contain up to 37 TM helices. To maximize the available structural repertoire, TM helices must cross each other at non-zero angles. However, with increasing distance from helix-helix crossing points, sidechains will lose interhelical contacts. Apparently, this downside is compensated by introducing helix kinks and by wedging either non-helical residues or additional helices into a helix-helix interface (Fig. 1). In contrast to wedges, helix kinks may be created by a single point mutation that introduces proline. The fusion of the proline sidechain to the backbone nitrogen atom and the loss of helical hydrogen bonding introduces a helix kink of varying severity^3^,^4^,^5^,^6^,^7^. Mutations to proline consequently may have played a central role in the evolution of membrane proteins.

Indirect support for this hypothesis is abundant. Inspection of membrane protein structures reveals that helix kinks are frequently centered around proline residues (Fig. 1a,b)^4^,^6^,^8^,^9^. Even for non-proline kinks, it is likely that a proline first initiated this conformation but became redundant when tertiary contacts solidified the kink conformation^10^. The important function of prolines further extends to preventing membrane protein misfolding^11^. Despite the benefit of prolines, their evolutionary origin is unclear as proline substitutions are difficult to establish. TM sequences from the Human Gene Mutation Database have one of the highest phenotypic incidences for proline substitutions^12^. Moreover, in the seven-helix bundle protein bacteriorhodopsin, 15 proline substitutions were examined and all were found to destabilize the protein^13^. Similarly, in the glycophorin A homodimer, proline scanning of the TM helix only destabilized the protein^14^. While protein stability may be recoverable by subsequent mutations, the extensive structural perturbations created by the sidechain geometry of proline invariably make such a pathway challenging. In comparison, an initially stabilizing kink followed by destabilizing, adaptive mutations appears more advantageous. Destabilizing mutations are abundant and therefore faster to occur in the critical time window after the initial mutation. Here, we provide experimental support for the second pathway to provide insight into the evolution and design principles of membrane proteins.

## Results and Discussion

In the family of integrin adhesion receptors, the TM complex between α and β subunits constrains the receptor in its inactive conformation^15^,^16^. Specifically, the inactive ectodomains and associated TM complex stabilize each other^15^. A substantial loss of αβ TM affinity and the ensuing TM complex dissociation allows the ectodomains to rearrange, thereby activating the receptor to bind ligands. For example, the proline substitution L718P in the TM helix of the β3 subunit is a disease-causing mutation in humans arising from spontaneous receptor activation^17^. This structural architecture of integrins makes the study of integrin αβ TM complexes in isolation relevant to understanding their allosteric regulation. In the integrin αIIbβ3 receptor, we previously discovered the ability of β3(A711P) to compensate the activating β3(K716A) substitution in an evolutionary selection screen^18^. If β3(A711P) indeed stabilizes the inactive receptor conformation, it must increase αIIbβ3 TM complex affinity by itself. Thus we determined the thermodynamic stability of the αIIbβ3(A711P) TM complex in phospholipid bicelles^19^ by isothermal titration calorimetry. We found a stabilization of −0.82 ± 0.01 kcal/mol relative to the wild-type TM complex stability, termed ΔG°_TM_, of −4.84 ± 0.01 kcal/mol (Table 1). Indeed, β3(A711P) is the first documented example of a stabilizing proline substitution in a membrane protein that we are aware of. It reveals that proline substitutions can increase the complexity of membrane proteins by directly stabilizing interhelical interactions.

**Figure 2:**
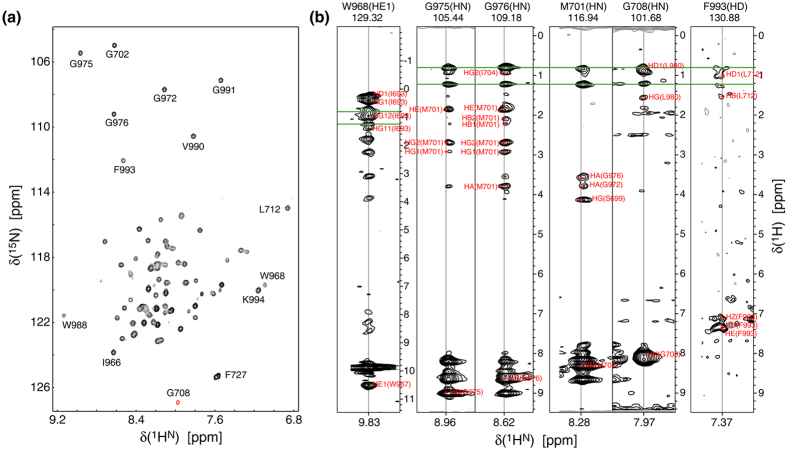
NMR spectra of the integrin αIIbβ3(A711P) TM complex. (**a**) TROSY-type H-N correlation spectrum of disulfide-linked ^2^H/^13^C/^15^N-αIIb(A963C)–^2^H/^13^C/^15^N-β3(G690C/A711P). (**b**) 3D NOESY-TROSY strips of disulfide-linked ^2^H/^15^N-αIIb(A963C)–β3(G690C/A711P) and αIIb(A963C)–^2^H/^15^N-β3(G690C/A711P) illustrate interhelical NOEs. NOEs to protonated lipids are indicated by green lines. All spectra were recorded at 40 °C and 700 MHz.

To understand the basis of β3(A711P), we determined the structure of the αIIbβ3(A711P) TM complex in isotropic phospholipid bicelles by multidimensional heteronuclear NMR spectroscopy. In the structure determination of the wild-type αIIbβ3 TM complex, we had used selectively methyl-labeled protein and deuterated lipids to obtain interhelical NOE distance restraints^15^. Upon inspecting this structure, we predicted that it is possible to detect a similar number of distance restraints by measuring NOEs between backbone ^1^H^N^ and sidechain ^1^H nuclei across the helix-helix interface. We thus combined one perdeuterated and one protonated subunit in protonated lipids. Additionally, as described previously^20^, we cross-linked the complex outside of the TM region by a disulfide bond to maximize the concentration of dimer, to suppress residual monomer signals and to improve dimer lineshapes. This approach permitted the detection of interhelical NOEs up to ^1^H^N^-^1^H^α^ pairs (Fig. 2), albeit only in the vicinity of glycines packed in the dimerization interface. The reduced range of ^1^H^N^-^1^H as opposed to ^1^H^CH3^-^1^H distances is mitigated by the high rigidity of backbone ^1^H^N^ nuclei compared to sidechain ^1^H^CH3^ nuclei. Moreover, it was further compensated by observing intersubunit NOEs to the indole ^1^H^N^ nuclei of αIIb(W968) and β3(W715), which are located at the N- and C-helix termini, and by detecting NOEs to the aromatic ring of αIIb(F993) in fractionally deuterated samples (Fig. 2). Membrane proteins show an abundance of aromatic residues in the membrane-water interface^21^,^22^, which makes the presented approach effective for the structure determination of membrane proteins with packed glycines in the presence of protonated lipids or detergents. Further structural restraints included H-N residual dipolar couplings collected for the perdeuterated complex. An ensemble of 20 structures was calculated by simulated annealing with a coordinate precision of 0.33 Å for backbone heavy atoms (Supplementary Figure 1 and Supplementary Table 1).

**Figure 3:**
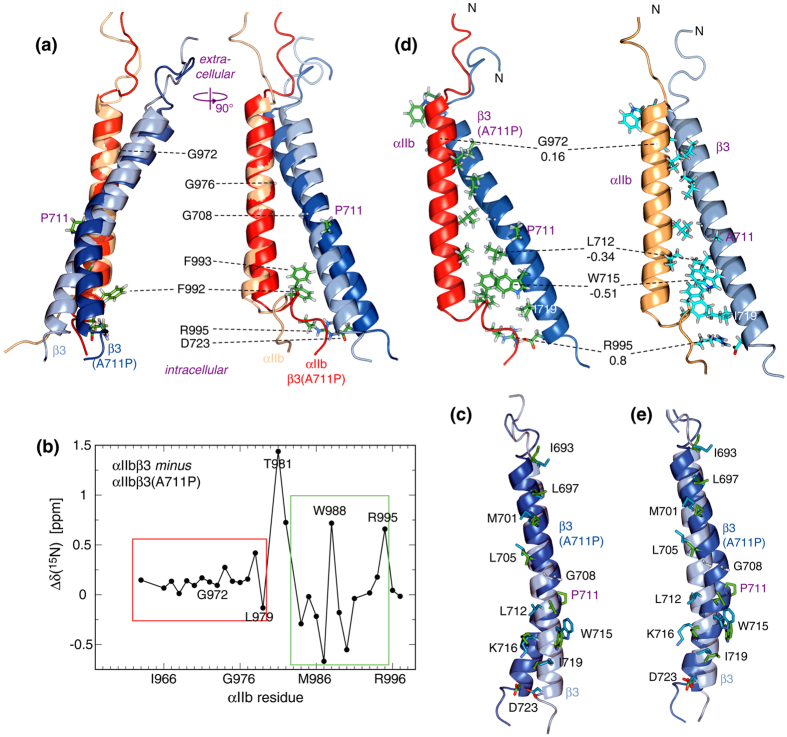
Structure of the integrin αIIbβ3(A711P) TM complex. (**a**) Comparison of integrin αIIbβ3(A711P) and αIIbβ3 TM complex structures. The structures were superimposed on the backbone heavy atoms of αIIb(W967-L979). (**b**) Chemical shift differences between αIIb backbone ^15^N nuclei of non-covalently linked αIIbβ3(A711P) and αIIbβ3 TM complexes. (**c**) Comparison of β3 sidechain orientations in the αIIbβ3(A711P) and αIIbβ3 TM complex structures. TM complex coordinates were superimposed as shown in panel *a*. (**d**) Comparison of αIIb(G972), β3(L712), β3(W715) and αIIb(R995) sidechain interactions between αIIbβ3(A711P) and αIIbβ3 TM complex structures. ΔΔG°,′ associated with the αIIb(G972A), β3(L712A), β3(W715Y) and αIIb(R995A) substitutions (Table 1) are indicated. (**e**) Comparison of β3 sidechain orientations when superimposing β3 backbone coordinates near the TM termini. PDB entries 2k9j (αIIbβ3) and 2n9y (αIIbβ3(A711P)) were used.

In the wild-type αIIbβ3 TM complex, two association motifs were differentiated^15^. The outer membrane clasp (OMC) is characterized by sidechain packing into the helix grooves created by αIIb(G972), αIIb(G976) and β3(G708). The inner membrane clasp (IMC) is characterized by the wedging of αIIb(Phe992-Phe993) to connect the separating TM helices and to maximize electrostatic αIIb(Arg995)-β3(Asp723) interactions (Fig. 3a). In the αIIbβ3(A711P) TM complex, these interactions were maintained albeit with changes. The ^15^N chemical shift differences between αIIb when complexed with either β3 or β3(A711P) illustrated that structural changes predominantly took place for IMC residues and residues that pack near the mutation site (Fig. 3b). The αIIb(W967-L979) helical segment was largely invariant, making it suitable to superimpose αIIbβ3 and αIIbβ3(A711P) coordinates to illustrate long-range structural differences. Within the dimer, the A711P substitution caused a 35 ± 2° kink in the β3 helix. The impact of this kink was minimized by maintaining αIIb interhelical packing against β3(G708) while distributing the changes in β3 helix directions to both the OMC and IMC (Fig. 3a). In the OMC, no significant rotation about the helix axis relative to wild type (swivel movement) took place (Fig. 3c). Changes in interhelical sidechain distances were apparently compensated by modifications of sidechain conformations (Fig. 3c). On the other hand, in the IMC changes in interhelical distance and swivel orientation were encountered. These changes altered αIIb contacts with β3 residues L712, W715, K716, I719 and D723 in the dimerization interface and increased towards the C-terminus (Fig. 3c).

To achieve a quantitative context for discussing changes in sidechain contacts, we determined changes in thermodynamic stabilities of four point mutations between the αIIbβ3(A711P) and αIIbβ3 TM complexes. Specifically, ΔΔG°,′ = (ΔG°_αIIb__β3,mutant_ −ΔG°_αIIb__β3_) − (ΔG°_αIIb__β3(A711P),mutant_ −ΔG°_αIIb__β3(A711P)_) was quantified to compare the disturbance created by a mutation relative to its respective αIIbβ3 and αIIbβ3(A711P) reference structure. In accordance with largely invariant OMC interactions, ΔΔG°,′ was small for αIIb(G972A) with 0.16 ± 0.03 kcal/mol (Fig. 3d and Table 1). In the IMC, the swivel for β3(L712) centered its sidechain more directly in the dimerization interface (Fig. 3c,d) and a ΔΔG°,′ of −0.34 ± 0.01 kcal/mol revealed improved sidechain packing. Interestingly, β3(W715) moved in such a way that its pyrrole ring position in αIIbβ3 was replaced by its benzene ring in αIIbβ3(A711P) (Fig. 3c). This swap heightened hydrophobic interactions with αIIb(Phe993) and, with ΔΔG°,′ = −0.51 ± 0.04 kcal/mol for β3(W715Y), contributed to TM complex stabilization. The swivel of the IMC helix segment of β3 rotated Lys716 towards the dimerization interface (Fig. 3c), which allows more favorable hydrogen bonding with αIIb(Phe992/CO) relative to the wild-type structure. Unfortunately, the strongly destabilizing nature of β3(K716) substitutions^18^ did not allow the direct quantification of ΔΔG°,′ at this site. β3(Ile719) engages in hydrophobic packing below the wedged aromatic rings and its increased distance from αIIb in the αIIbβ3(A711P) TM complex is expected to be destabilizing (Fig. 3d). Likewise, the increased distance of β3(D723) from αIIb requires an adjustment of the αIIb backbone conformation to make electrostatic contacts with αIIb(R995) (Fig. 3d). ΔΔG°,′ of 0.8 ± 0.2 kcal/mol for αIIb(R995A) confirmed the destabilizing nature of this adjustment.

As is the case with β3(A711P), prolines in membrane protein structures are frequently encountered near the center of TM helices^4^,^22^,^23^. Based on the αIIbβ3(A711P) TM complex structure, we propose a general scheme for incorporating proline kinks in membrane proteins: maintain interhelical packing close to the proline kink and predominantly repack either the helix segment preceding or succeeding the kink. In case of αIIbβ3, the OMC with glycine packing interactions was largely maintained (Fig. 3a,b), which is likely of general validity due to the high structural specificity of this interaction. With respect to β3(G708), A711P created a GXXP motif. Proline generally kinks away from the H-bond that is lost (Fig. 3c)^4^, which makes the GXXP spacing well suited for heterodimeric helix-helix packing. In the repacked helix segment, the increasing separation of interhelical interactions tends to diminish interhelical contacts. To achieve a net stabilization of helix-helix interactions, contacts that remain within sidechain packing distances must be optimized and, evidently, the gain in stability must supersede the destabilization from compromised sidechain contacts. In case of αIIbβ3(A711P), interactions within two helix turns C-terminal to the proline substitution were optimized (Fig. 3d and Table 1). Additionally, based on the β3(A711P)-induced chemical shift changes of αIIb (Fig. 3b), favorable contributions from any repacking of β3(G708) with αIIb(L980) cannot be excluded.

The alternative to maintaining interhelical contacts near the proline kink would be to preserve interactions at the TM helix termini. When inspecting this possibility for αIIbβ3(A711P), it is apparent that mostly αIIb(R995)-β3(D723) benefits whereas packing on β3(G708) and αIIb(G976) would be less intimate (Fig. 3e). This mode of interaction appears generally inferior as it creates a packing void at the β3 helix centre that is difficult to fill even when more TM helices were to be added. Despite the relatively complex and extensive packing of the integrin αIIbβ3 TM complex (Fig. 3), β3(A711P) revealed that it is not as well packed as possible. This is perhaps not surprising for two reasons. First, to accomplish the allosteric regulation of the receptor, ΔG°_TM_ must be balanced with the affinity of intra- and extracellular receptor agonists and with the stability of the inactive versus the active ectodomains^15^,^24^. Secondly, the increase in ΔG°_ΤΜ_ came at the expense of αIIb(R995)-β3(D723) destabilization. This interaction is disrupted during talin-mediated integrin activation^25^. With its reduced importance for TM complex stability in αIIbβ3(A711P), talin is now unable to activate the receptor in its presence^26^. In sum, we have revealed the structural and thermodynamic requirements for incorporating proline into TM helix-helix interactions and gained insight into constraints that underlie the evolution of such kinks.

## Methods

### NMR spectroscopy

The disulfide-linked αIIb(A963C)–β3(G690C/A711P) dimer was prepared applying published protocols^20^ and incorporated human integrin sequences αIIb(A958-P998) and β3(P685-F727) with β3(C687S). Perdeuterated peptides were produced using 99% d_7_-glucose, 99% ^15^ND_4_Cl and 99% D_2_O. A fractionally deuterated ^2^H/^13^C/^15^N-αIIb(A963C)–β3(G690C/A711P) sample was prepared by growing *E. coli* cells in 60% D_2_O using protonated precursors. Freeze-dried peptide was reconstituted in 320 μL of 350 mM 1,2-dihexanoyl-*sn*-glycero-3-phosphocholine (DHPC), 105 mM 1,2-dimyristoyl-*sn*-glycero-3-phosphocholine (DMPC), 6% D_2_O, 0.02% w/v NaN_3_ buffered by either 25 mM NaH_2_PO_4_/Na_2_HPO_4_, pH 7.4 or 25 mM HEPES·NaOH, pH 7.4 for a final concentration of 0.8 mM and bicelle q-factor of 0.3.

Starting from the ^1^H^N^, ^15^N, ^13^C^α^, ^13^C^β^, and ^13^C′ assignment of the αIIbβ3 TM complex and the β3(A711P/K716A) TM segment^15^,^18^, backbone assignments of ^2^H/^13^C/^15^N-αIIb(A963C)–^2^H/^13^C/^15^N-β3(G690C/A711P) were achieved employing HNCA, HNCO, HNCACB and NOESY-TROSY experiments. ^15^N-edited NOESY-TROSY experiments using ^2^H/^15^N-αIIb(A963C)–β3(G690C/A711P) or αIIb(A963C)–^2^H/^15^N-β3(G690C/A711P) dimers were acquired with mixing times of 120, 150 and 175 ms. Using [60% ^2^H]/^13^C/^15^N-αIIb(A963C)–β3(G690C/A711P), an aromatic ^13^C-edited NOESY-HSQC experiment (mixing time 150 ms) was recorded. Sidechain assignments started again from the αIIbβ3 TM complex and were similar to the aforementioned NOESY spectra. In a general case, NOESY experiments for ^2^H/^14^N-α–^1^H/^15^-β and ^1^H/^15^N-α–^2^H/^14^-β can establish sidechain assignments in combination with standard experiments. Sidechain and NOE assignments were carried out manually using the program CARA. H-N residual dipolar couplings (RDC) were measured twice in compressed polyacrylamide gels (scalar product 0.983) using ^2^H/^15^N-αIIb(A963C)–^2^H/^15^N-β3(G690C/A711P) dimer^20^. All NMR experiments were carried out on a cryoprobe-equipped Bruker Avance 700 spectrometer at 40 °C.

### Structure calculation of the integrin αIIbβ3(A711P) TM complex

Structure calculations were carried out by simulated annealing, starting at 3000 K using the program XPLOR-NIH^27^. Backbone torsion angle restraints were extracted from ^15^N, ^13^C^α^, ^13^C^β^, and ^13^C′ chemical shift patterns^28^. Within experimental uncertainties, H-N RDCs measured for the αIIbβ3(A711P) TM dimer fitted the αIIb and β3(A711P/K716A) TM monomer structures^20^. This congruence permitted the use of H-N, C^α^-C′, N-C′ RDCs measured for these monomers to further restrict the individual αIIb and β3(A711P) backbone conformations. An employed torsion angle potential of mean force^29^ was biased to use the experimental χ_1_ angles detected in the monomeric αIIb and β3(A711P/K716A) TM segments, which mostly corresponded to their default values. Moreover, the sidechains of αIIb(Phe992) and β3(Lys716) were adjusted to snorkel. Aside from standard force field terms for covalent geometry (bonds, angles, and improper dihedrals) and nonbonded contacts (Van der Waals repulsion), dihedral angle restraints were implemented using quadratic square-well potentials. In addition, a backbone-backbone hydrogen-bonding potential was employed^30^. A quadratic harmonic potential was used to minimize the difference between predicted and experimental residual dipolar couplings (RDC; Δ^1^*D*). The final values for the force constants of the different terms in the simulated annealing target function were as previously described^15^. Supplementary Table 1 summarizes the structural statistics for all 20 calculated structures. The structures together with the energy-minimized average structure and structural constraints have been deposited in the Protein Data Bank and BMRB with accession numbers 2n9y and 25920, respectively.

### Isothermal titration calorimetry

ITC measurements of the peptides listed in Table 1 were carried on a Microcal VP-ITC calorimeter. 10 μM of β3 peptide in the 1.425 ml sample cell was titrated with αIIb peptide by injecting 9 μl aliquots over a period of 10 s each. Measurements were carried out in 43 mM 1,2-dihexanoly-*sn*-glycero-3-phosphocholine (DHPC), 17 mM 1-palmitoyl-2-oleoyl-*sn*-glycero-3-phosphocholine (POPC), 25 mM NaH_2_PO_4_/Na_2_HPO_4_ pH 7.4 at 28 °C. Prior to data analysis, the measurements were corrected for the heat of dilutions of the αIIb and β3 peptides. The αIIbβ3 complex stoichiometry was fixed at 1:1^31^ and the reaction enthalpy (ΔH°) and K_XY_ were calculated from the measured heat changes, δH_i_, as described previously^31^. The entropy change, ΔS°, is obtained as (ΔH°–ΔG°)/T.

## Additional Information

**How to cite this article**: Schmidt, T. *et al*. Structural and thermodynamic basis of proline-induced transmembrane complex stabilization. *Sci. Rep.* **6**, 29809; doi: 10.1038/srep29809 (2016).

## Supplementary Material

Supplementary Information

## Figures and Tables

**Figure 1:**
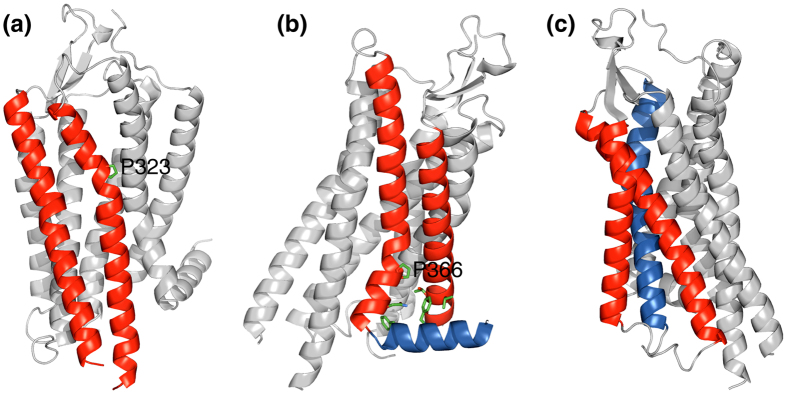
Transmembrane helix-helix interfaces in the neurotensin receptor 1. (**a**) Proline kink-mediated helix-helix packing. (**b,c**) Wedging of either non-helical residues or an additional helix into a helix-helix interface. PDB entry 4bwb was used^33^.

**Table 1:** Thermodynamic stability of mutant αIIbβ3 TM complexes.

Peptides	K_XY_^a^	ΔH° [kcal/mol]	ΤΔS° [kcal/mol]	ΔG° [kcal/mol]	ΔΔG°^,′^^b^ [kcal/mol]
αIIb + β3^c^	3250 ± 60	−16.0 ± 0.1	−11.1 ± 0.1	−4.84 ± 0.01	—
αIIb + β3(A711P)	12700 ± 200	−16.9 ± 0.1	−11.2 ± 0.1	−5.66 ± 0.01	—
αIIb(G972A) + β3	1080 ± 30	−14.2 ± 0.2	−10.1 ± 0.2	−4.18 ± 0.01	—
αIIb(G972A) + β3(A711P)	5500 ± 300	−16.2 ± 0.3	−11.0 ± 0.3	−5.16 ± 0.03	0.16 ± 0.03
αIIb + β3(L712A)	1900 ± 50	−12.0 ± 0.1	−7.4 ± 0.1	−4.52 ± 0.01	—
αIIb + β3(A711P/L712A)	4200 ± 100	−12.8 ± 0.1	−7.8 ± 0.1	−5.00 ± 0.01	−0.34 ± 0.01
αIIb + β3(W715Y)	1300 ± 40	−14.2 ± 0.2	−9.9 ± 0.2	−4.30 ± 0.02	—
αIIb + β3(A711P/W715Y)	2200 ± 100	−14.4 ± 0.4	−9.8 ± 0.4	−4.61 ± 0.03	−0.51 ± 0.04
αIIb(R995A) + β3^c^	250 ± 70	−15 ± 4	−12 ± 4	−3.3 ± 0.2	—
αIIb(R995A) + β3(A711P)	4000 ± 300	−5.6 ± 0.2	−0.59 ± 0.2	−4.98 ± 0.04	0.8 ± 0.2

^a^Measurements performed in 43 mM DHPC, 17 mM POPC, 25 mM NaH_2_PO_4_/Na_2_HPO_4_ pH 7.4 solution at 28 °C (effective bicelle q-factor of 0.5).

^b^ΔΔG°,′ = (ΔG°_αIIb__β3,mutant_ −ΔG°_αIIb__β3_) − (ΔG°_αIIb__β3(A711P),mutant_ −ΔG°_αIIb__β3(A711P)_)

^c^Measured previously by competitive binding experiments^32^, resulting in larger experimental uncertainties than direct measurements.
