# Supplementary material for: Structural and thermodynamic basis of proline-induced transmembrane complex stabilization
Source: Sci Rep. 2016 Jul 20;6:29809. doi: 10.1038/srep29809 (PMC4951694; doi:10.1038/srep29809)
Supplement: Supplementary Information [file srep29809-s1.pdf]

SUPPLEMENTARY INFORMATION

Structural and Thermodynamic Basis of Proline-induced Transmembrane Complex Stabilization

Thomas Schmidt, Alan J. Situ, and Tobias S. Ulmer

Department of Biochemistry & Molecular Biology and Zilkha Neurogenetic Institute, Keck School of Medicine, University of Southern California, 1501 San Pablo Street, Los Angeles, CA 90033, USA

TABLE OF CONTENTS

Supplementary Figure S1. Superposition of integrin αIIbβ3(A711P) TM complex structures 2

Supplementary Table S1. Structural statistics for integrin αIIbβ3(A711P) TM complex 3

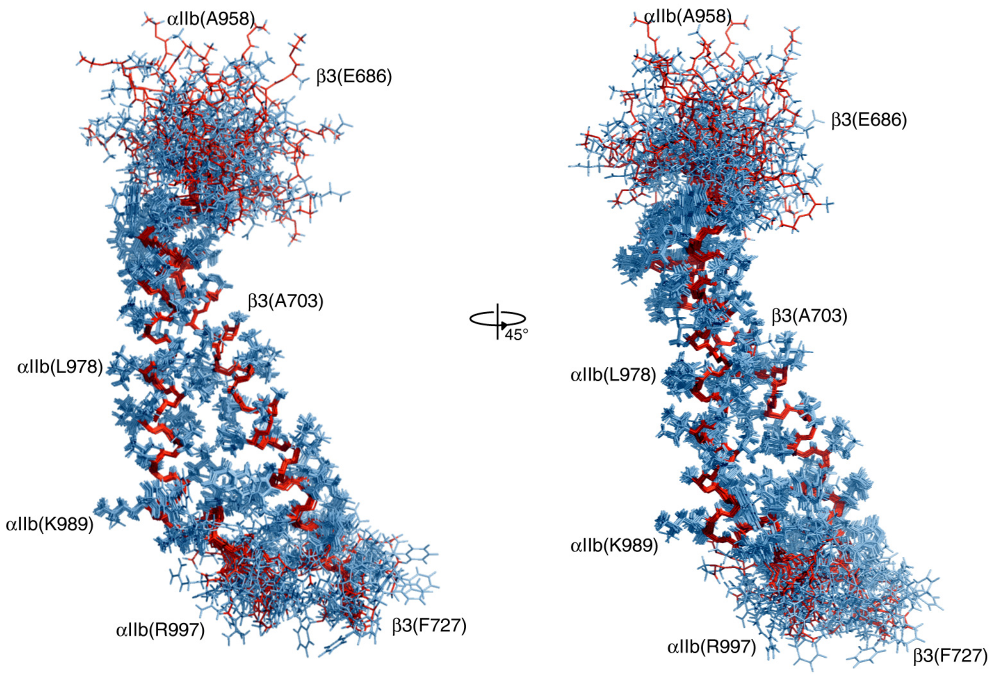

Supplementary Figure S1. Superposition of all 20 calculated simulated annealing structures of the integrin αIIbβ3(A711P) TM complex. The backbones and sidechains are shown in *red* and *blue*, respectively.

Supplementary Table S1. Structural statistics for the integrin αIIbβ3(A711P) TM complex[a]

R.m.s. deviations from experimental dihedral restraints (deg)	
All *(155)*[b]	0.6 ± 0.1
R.m.s. deviations from experimental residual dipolar couplings (Hz)[c]	
$^{1}D_{NH}$ *(30)*, *αIIb(A963C)–β3(G690C/A711P)*	1.02 ± 0.04
$^{1}D_{NH}$ *(30)*, *αIIb(A963C)–β3(G690C/A711P)*	0.57 ± 0.03
$^{1}D_{NH}$ *(30)*, *αIIb*	0.98 ± 0.03
$^{1}D_{NC'}$ *(29)*, *αIIb*	0.85 ± 0.04
$^{1}D_{C_{\alpha}C'}$ *(30)*, *αIIb*	0.95 ± 0.04
$^{1}D_{NH}$ *(30)*, *β3(A711P,K716A)*	1.96 ± 0.09
$^{1}D_{NC'}$ *(29)*, *β3(A711P,K716A)*	1.90 ± 0.08
$^{1}D_{C_{\alpha}C'}$ *(30)*, *β3(A711P,K716A)*	1.85 ± 0.11
R.m.s. deviations from experimental distance restraints (Å)	
All (*163*)	0.06 ± 0.01
Intraresidue (*4*)	0.01 ± 0.01
Interresidue sequential ($\|i - j\| = 1$) (*59*)	0.03 ± 0.01
Interresidue short range ($1 < \|i - j\| < 5$) (*69*)	0.07 ± 0.01
Interresidue long range ($\|i - j\| \geq 5$) (*31*)	0.08 ± 0.01
Deviations from idealized covalent geometry	
Bonds (Å)	0.003 ± 0.000
Angles (deg)	0.59 ± 0.02
Impropers (deg)	0.54 ± 0.01
Coordinate precision (Å)[d]	
Backbone non-hydrogen atoms	0.33
All non-hydrogen atoms	0.70
Measures of structural quality	
E_{LJ} (kcal mol^{-1})[3] [e]	-311.9
Residues in most favorable region of Ramachandran plot[f]	99.5%

[a]Statistics for all 20 calculated simulated annealing structures, encompassing structured residues αIIb(I966-R995) and β3(I963-D723).

[b]Torsion angle restraints included 64 ϕ, 64 ψ, and 27 χ_1 angles.

[c]R.m.s. deviations are normalized to an alignment tensor magnitude of 10 Hz.

[d]Defined as the average r.m.s. difference between the 20 simulated annealing structures and the mean coordinates.

[e]The Lennard–Jones van der Waals energy was calculated with the CHARMM PARAM 19/20 parameters and not included in the simulated annealing target function.

[f]Calculated using PROCHECK V3.4.4 (Laskowski RA, Macarthur MW, Moss DS, Thornton JM. Procheck - A Program To Check The Stereochemical Quality Of Protein Structures. J Appl Crystallogr 26, 283-291 (1993)).
